# CAD/CAM nitinol bonded retainer versus a chairside rectangular-chain bonded retainer: A multicentre randomised controlled trial

**DOI:** 10.1177/14653125221118935

**Published:** 2022-09-04

**Authors:** Adam C Jowett, Simon J Littlewood, Trevor M Hodge, Harmeet K Dhaliwal, Jianhua Wu

**Affiliations:** 1 Orthodontic Department, Leeds Dental Institute, Leeds, UK; 2 Orthodontic Department, St Luke’s Hospital, Bradford, UK; 3 University of Leeds, School of Dentistry, Leeds, UK

**Keywords:** Retention, Retainers, Stability, Failure, Patient satisfaction, Randomised controlled trial

## Abstract

**Background:**

Bonded retainers are widely used to maintain the positions of anterior teeth after orthodontic treatment. Various types of bonded retainer exist however, there is a lack of evidence to indicate which type is superior.

**Aim:**

To compare upper and lower CAD/CAM nitinol bonded retainers (Memotain^®^) with upper and lower chairside rectangular-chain bonded retainers (Ortho-FlexTech™), in terms of stability, retainer failures and patient satisfaction.

**Trial design:**

Multi-centre, two-arm, parallel-group, randomised controlled clinical trial with 1:1 allocation.

**Setting:**

Three trial centres: University Teaching Hospital; District General Hospital; and Specialist Orthodontic Practice. All treatment was provided free as part of a state-funded healthcare system.

**Materials and methods:**

A total of 68 patients were randomly allocated to receive either upper and lower Memotain^®^ bonded retainers or upper and lower Ortho-FlexTech™ bonded retainers. Ten trained operators placed and reviewed the bonded retainers. Measurements were carried out on study models taken at debond and after six months. Patient satisfaction questionnaires were completed at six months following debond.

**Results:**

The trial was terminated due to the high number of failures (50%) of the upper Memotain® retainers within six months. Memotain^®^ retainers were three times more likely to fail (unadjusted hazard ratio = 2.82, 95% confidence interval = 1.00-7.99) than Ortho-FlexTech™ retainers at six months in the upper arch. Patients were satisfied with both types of retainer.

**Limitations:**

Early termination of the trial means that the a priori sample size was not reached, so outcomes should be interpreted with caution.

**Conclusion:**

The trial was terminated early due to the high failure rate of upper Memotain^®^ bonded retainers. They had a higher risk of failure in the maxillary arch when compared to upper Ortho-FlexTech™ bonded retainers after six months.

## Introduction

Maintaining a stable result after orthodontic treatment remains one of the great challenges in orthodontics. Changes in tooth position after orthodontic treatment can arise as a result of relapse, defined as a return of teeth towards their original position. Unwanted post-treatment changes could also be a consequence of normal age changes (Abdulraheem et al., 2020), which can lead to a reduction in arch length and perimeter, and a decrease in intercanine width, resulting in crowding and irregularity, particularly in the lower labial segment. Long-term studies of patients who stopped wearing their retainers after 1–2 years seem to show an inevitable and unpredictable level of unwanted post-treatment changes. In some cases, these changes were so severe that another course of orthodontic treatment was indicated (Little et al., 1981, 1988). This has resulted in an increasing number of clinicians recommending long-term retention to resist relapse (Littlewood et al., 2017).

There is a shortage of high-quality evidence to clearly indicate which is the best type of retention regimen to use for long-term retention (Al-Moghrabi et al., 2021; Littlewood et al., 2016). Randomised controlled trials comparing fixed retainers and removable retainers have not conclusively shown one type of retainer to be better than the other (Al-Moghrabi et al., 2018; Forde et al., 2018; Krämer et al., 2020, 2021; Storey et al., 2018). In one randomised trial it was shown that after four years, adherence with a removable retainer had significantly dropped, resulting in better retention with fixed retainers in the lower arch (Al-Moghrabi et al., 2018). Patients have expressed a preference for bonded retainers due to the fact that they do not need to remember to wear them (Forde et al., 2018; Krämer et al., 2020, 2021).

As the long-term benefits of long-term retention have been recognised, there has been an increased use of bonded retainers. However, it is important to recognise that they do have potential complications (Kučera et al., 2021). These include: failure (Kocher et al., 2019); potential for adverse effect on periodontal health (Storey et al., 2018; Tacken et al., 2010); unwanted tooth movement with the retainer in situ (Kučera and Marek, 2016); and adverse effects on general health (Eliades et al., 2011).

Failures of bonded retainers can occur as a result of debonding of the composite from the enamel, failure between the wire and the composite or fracture of the retainer (Kučera et al., 2021). The most common failure occurs between the adhesive and the enamel (Dahl and Zachrisson, 1991; Forde et al., 2018), which may be the result of poor clinical technique (in particular, lack of moisture control during bonding). Composite does not bond chemically to wire retainers, so this bond relies on mechanical retention between the composite and the surface of the wire. The likelihood of wire fracture depends on the type and diameter of wire used, and whether there is repeated stress on the wire, as a result of direct occlusal trauma from the opposing arch or repeated flexing of the wire (Kučera et al., 2021).

The aetiology of unwanted tooth movement with a bonded retainer still in situ is poorly understood but could be due to a tooth-moving force from within the wire, or a force inadvertently applied by the clinician or the patient. It has been suggested that there may be inherent activity in the archwire, which may be placed in an active position during bonding, or distorted due to occlusal contacts or patient habits (Kučera et al., 2021). It is unclear which is the best type of bonded retainer to minimise these various complications, while also offering reliable retention and being both comfortable and acceptable to patients.

In recent years, two new fixed retainers have entered the market, Memotain^®^, a CAD/CAM nitinol retainer, and Ortho-FlexTech™, a rectangular-chain retainer that can be directly bonded at the chairside.

Memotain^®^ is laser-cut from a nitinol sheet or blank, so no bending of the wire is required. The bending sites are thought to be sites of increased risk of wire fracture, so the Memotain^®^ method of manufacture aims to eliminate this (Kravitz et al., 2017). Once cut, the wire is electropolished in an ion-charged bath that smooths, cleans and polishes the wire, increasing corrosion resistance and reportedly making the wire less susceptible to microbial colonisation. This process also aims to round off the corners of the square wire potentially increasing patient comfort. To manufacture the Memotain^®^ retainer, a detailed record of the lingual/palatal surfaces of the teeth in the form of a study model or 3D scan is required. An opposing model and a record of the occlusion is required to allow the digital positioning of upper retainers away from any occlusal interferences. The effectiveness of Memotain^®^ was recently assessed in a randomised controlled clinical trial, undertaken in a university clinic, comparing it with five-strand co-axial stainless-steel wires in the lower arch. This study showed no difference in periodontal outcome or survival rates over six months (Kartal et al., 2021). In another university-based randomised controlled clinical trial investigating retention of the lower labial segment, Memotain^®^ retainers were compared with multi-stranded stainless-steel twistflex wire, a single-stranded nickel-free titanium bonded wire and vacuum-formed removable retainers (Alrawas et al., 2021). The team could find no significant difference in clinical failure rate between any of the retainers. A third university-based randomised controlled trial also investigated the performance of the Memotain^®^ retainer in the lower arch. They found there was no difference in periodontal or relapse outcomes compared with other bonded retainers (Adanur-Atmaca et al., 2021). None of these randomised controlled trials investigated Memotain^®^ in the upper arch.

Ortho-FlexTech™ (Reliance Orthodontic Products, Itasca, IL, USA) is a 0.039 × 0.014-inch chairside rectangular-chain bonded retainer available in stainless steel or 14-carat white gold (etched and non-etched versions). The retainer is measured and fitted chairside, requires no laboratory input and offers the potential for quick easy and economical placement. Ortho-FlexTech™ has gained in popularity over recent years partly due to its ease of adaptability and direct placement properties (Padmos et al., 2018; Patel et al., 2017).

This study is designed to compare upper and lower Memotain^®^ bonded retainers with Ortho-FlexTech™ bonded retainers.

The primary aim of the present study was to compare stability, measured in terms of alignment of the upper and lower labial segments and maintenance of intercanine widths. The secondary aims were to compare the following: failure rates for each retainer; patient satisfaction; and performance of each retainer in different settings (University Teaching Hospital, District General Hospital, and Specialist Orthodontic Practice) and with different grade operators (Consultant Orthodontist, Orthodontic Registrar and Orthodontic Therapist).

The null hypotheses were as follows: there is no difference in the maintenance of the intercanine width or the alignment of teeth bonded with Memotain^®^ and Ortho-FlexTech™ retainers in both the upper and lower arches; there is no difference in failure rates of Memotain^®^ and Ortho-FlexTech™ retainers in both the upper and lower arches; there is no difference in patient satisfaction with both Memotain^®^ and Ortho-FlexTech™ retainers in both the upper and lower arches; and the performance of the retainer is not affected by the setting or the grade of clinician placing the retainer.

## Methods

### Study design and ethical approval

This study was a multi-centre, multi-operator, prospective, two-arm, parallel-group, assessor-blinded, randomised controlled trial with 1:1 allocation. The study was conducted in accordance with the ethical principles outlined in the 1964 Declaration of Helsinki and its later amendments or comparable ethical standards. It received ethical approval by the Health Research Authority in May 2017 (IRAS reference: 185443) and by the Yorkshire and Humber Research Ethics Committee in July 2017 (REC reference: 16/YH/0463)

### Participants

Consecutive patients, nearing the completion of fixed appliance therapy, who required retainers were invited to take part in the trial. Participants were recruited from the orthodontic departments of one University Teaching Hospital (Leeds Dental Institute), one District General Hospital (St Luke’s Hospital, Bradford) and one primary care Specialist Orthodontic Practice (Beverley Orthodontic Centre). The retainers were placed by clinicians of different grades (Consultant Orthodontists, Orthodontic Registrars and Orthodontic Therapists). Treatment was provided for free under the UK’s National Health Service.

The following inclusion criteria were applied: had undergone a course of upper and lower fixed appliance orthodontic treatment with satisfactory correction of the presenting malocclusion; had a size and shape of anterior teeth that allowed placement of a bonded retainer; no missing anterior teeth in the upper and lower labial segments; brushed their teeth at least twice per day (as determined by questioning the patient); was in good health; was willing and able to comply with the trial regime; and had given written informed consent.

Participants may have presented with any malocclusion before orthodontic treatment and may have been managed on an extraction (premolar or molar) or a non-extraction basis. Participants may have been treated with removable or functional appliances in conjunction with their fixed orthodontic appliance treatment.

The following exclusion criteria were applied: cleft palate and/or other severe facial deformities; nickel allergy; poor periodontal health at the pre-debond appointment, including the presence of supragingival or subgingival calculus, or periodontal pocketing greater than 3.5 mm, as determined by a basic periodontal examination (BPE) probe; gross or uncontrolled caries; prosthodontic requirement in the upper or lower arch at the end of treatment; and a starting malocclusion that required extreme transverse correction (rapid maxillary expansion).

## Interventions and comparisons

Consecutive patients nearing the end their fixed appliance phase of orthodontic treatment, who fulfilled the inclusion criteria, were invited to take part in the trial. Initial eligibility screening was carried out by one of the operating clinicians. All potential participants were treated under Consultant supervision in one of the three trial centres. For non-English speaking patients, an interpreter was used to explain the research trial and to ask the patient if they wish to participate. This approach was continued throughout their review appointments. At the end of active treatment, participants were randomised into one of two groups and operators placed either upper and lower Memotain^®^ bonded retainers (Group 1) or upper and lower Ortho-FlexTech™ bonded retainers (Group 2) following a standardised operating procedure.

## Group 1: Memotain^®^

**Figure 1.**
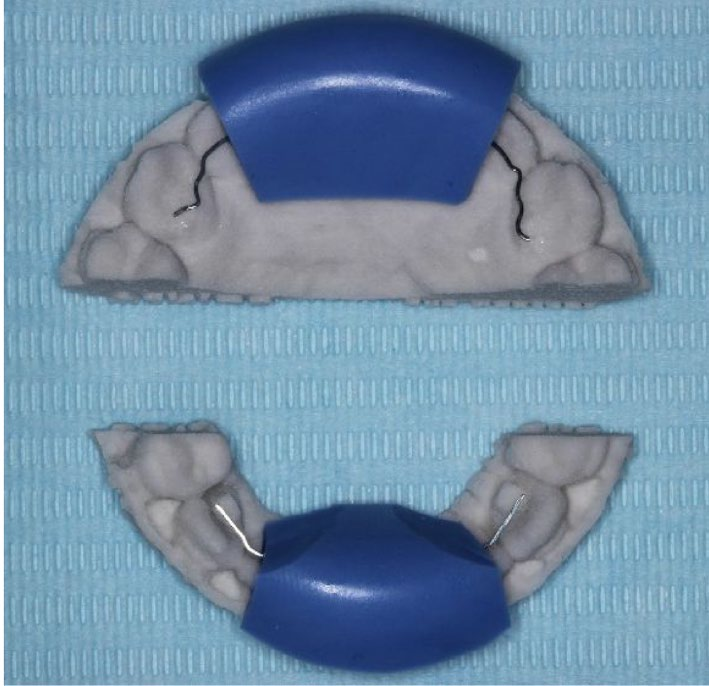
Memotain^®^ 3D printed sectional model, laser cut nickel-titanium retainer material and blue silicone transfer jig.

At the visit before debond, an impression or scan was taken of the palatal/lingual surfaces of the upper and lower labial segments. A record of the occlusion was also provided so that the upper retainer could be positioned in such a way as to minimise occlusal interferences. If the manufacturers had any questions about the position of the retainer, for example, a risk of occlusal interference as shown on the digital plan, the clinician was contacted. Once manufactured, the custom retainer, sectional 3D printed model and silicone transfer jig were sent to the clinician for fitting (Figure 1). The process of manufacturing and shipping of the retainers took approximately ten days.

## Group 2: Ortho-FlexTech™

These participants received a rectangular-chain bonded retainer formed from etched white carat gold. This was fitted directly at the chairside and shaped to lie passively against the lingual surface of the upper and lower canines and incisors. Either a pre-debond model or the patient’s own teeth were used to measure accurately an appropriate length of material to be bonded to the teeth. Participants randomised to Ortho-FlexTech™ did not require any pre-debond scans or impressions.

## Placement

A standard operating procedure for placement of Memotain^®^ and Ortho-FlexTech™ bonded retainers was agreed among the clinicians involved in the trial based on the manufacturer’s instructions for each product. Two 5-minute videos were produced explaining the individual stages involved in placing each of the two different retainers to standardise the operating procedure for all clinicians in the trial.

The standard operating procedure for both retainer materials involved the use of separate etch and bond alongside Transbond LR (3M Unitek, Monrovia, CA, USA) resin-based composite:

1. Removal of any hardened pellicle with a debond bur or sandblaster.
2. Prophy teeth with an oil-free pumice.
3. Wash with water for 30 s.
4. Isolate teeth and air dry.
5. Etch for 30 s.
6. Spray water for 30 s.
7. Dry with air from the 3-in-1 syringe for 15 s.
8. Apply primer, Transbond LR (3M Unitek, Monrovia, CA, USA) resin-based composite and light cure for 30 s:
  - Memotain^®^ – seat retainer with the provided jig, bond canines first, remove jig, then repeat stages 5–8 for incisors.
  - Ortho-FlexTech™ – place resin-based composite on all anterior teeth, seat retainer, light cure and then place resin-based composite over the top.
9. Check occlusion with articulating paper.

**Figure 2.**
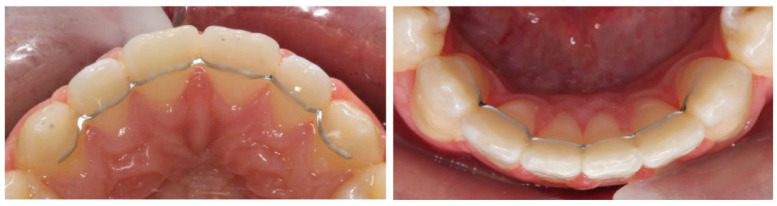
Fitted bonded retainers for participants randomised to Group 1: Memotain^®^.

**Figure 3.**
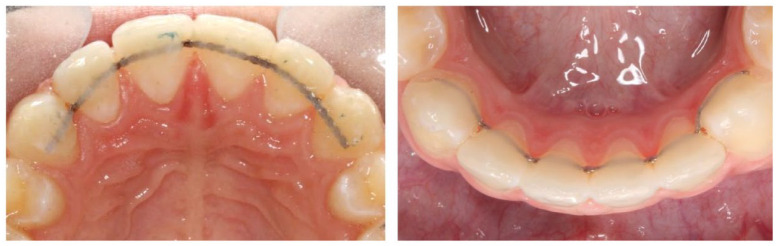
Fitted bonded retainers for participants randomised to Group 2: Ortho-FlexTech™.

After fitting the upper and lower bonded retainers, the upper and lower fixed appliances were removed. Figure 2 and Figure 3 show examples of participants recruited to Memotain® and Ortho-FlexTech™ respectively.

The decision was made not to use any additional clear plastic retainers, so that any relapse could be related to the performance of the bonded retainers, and not masked by the additional retention provided by a removable retainer.

## Outcomes

The primary outcome for the trial was stability of the intercanine width and alignment of the labial segments.

Secondary outcomes were failure rate, patient satisfaction and cost-effectiveness. Cost-effectiveness was included in the initial protocol but is not reported here.

Stability was measured by changes in Little’s Irregularity Index and intercanine width. All measurements were carried out on orthodontic study models using impressions taken at the debond and six-month review appointments. All measurements of stability were carried out with the aid of magnifying loupes and accompanying illumination to ensure maximum accuracy of measurements.

Before data collection, intra-rater reliability was assessed by measuring a sample of 20 sets of study models (10 upper models and 10 lower models) and repeating these measurements 28 days later. A total of 20 sets of study models (10 debond models and 10 six month models) were assessed by a specialist orthodontist (SD) not involved in the trial. Study model assessment was carried out on two separate occasions four weeks apart and the models were steam cleaned to remove any pencil marks in-between assessments. Intra-rater reliability was assessed using the intraclass correlation coefficient (ICC). Mean difference and standard deviation was carried out using a one-sample *t* test.

## Survival

Retainer survival was recorded as the time to the first episode of failure. A failure was defined as follows: bond failure between the composite and the enamel; bond failure between the wire and composite; fracture of the wire; and complete detachment from all the teeth.

The date of failure was recorded as the day the patient became aware of the problem, or alternatively, the date the clinician noted the failure (when participants were unaware of the failure).

## Patient satisfaction

Patient satisfaction was determined by a questionnaire based on the most relevant questions used in a similar study comparing maxillary and mandibular vacuum-formed retainers with maxillary and mandibular bonded retainers (Storey et al., 2018). Patient satisfaction questionnaires were handed to the patient to complete privately and anonymously. The questionnaires were then collected afterwards and identified using their unique research number.

The following Yes/No questions were asked for upper and lower retainers:

1. Did your upper retainer keep your teeth straight?
2. Was your upper retainer easy to look after?
3. Was your upper retainer comfortable?
4. Did your upper retainer affect your speech?
5. Did your upper retainer cause a problem that meant you needed to see your orthodontist?

The questionnaire was completed after six months.

## Sample size

The sample size was determined based on the primary objective of comparing the efficacy of bonded retainers in minimising the post-orthodontic treatment change in the arch alignment. A total of 42 participants were required in each group to achieve 90% power to detect a minimum clinical difference of 0.5 mm in Little’s Irregularity Index between the two groups with a known standard deviation of 0.7 mm (based on the relapse data from previous studies) (O’Rourke et al., 2016; Rowland et al., 2007) and with a significance level of 5% using a two-sided, two-sample *t* test. To allow for a potential dropout rate of 20% and to increase the sample size for the secondary outcome measurements, the planned sample size was increased to 50 per group resulting in a total of 100 participants (50 in each arm of the trial).

## Randomisation

The randomisation website (www.sealedenvelope.com) was used to allocate participants to treatment Group 1 or Group 2. This online randomisation tool involves setting up a mixed block size (2,4 and 6) randomisation list. Randomisation is carried out on the sealed envelope website using a trial specific password. As soon as anonymised participant details are entered, details of the group allocation is given and the primary researcher notified by email. This method aimed to streamline the randomisation process across multiple sites whilst making trial recruitment as simple as possible. The system was set up prior to trial commencement and any details of the randomisation list permanently deleted following set up.

## Blinding

**Figure 4.**
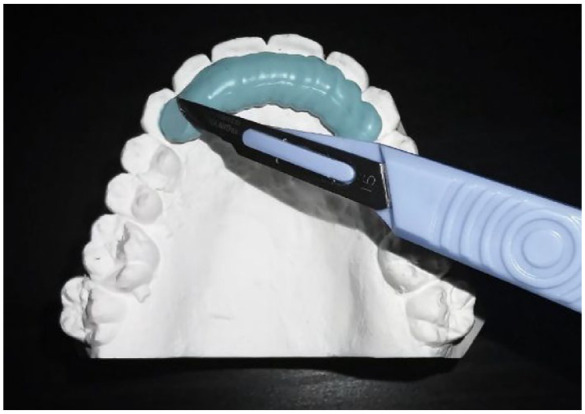
The assessor was blinded to the type of retainer by covering the retainer with Blu-Mousse.

It was not possible to blind the operator or patient to the type of retainer used, but to blind the assessor, a novel technique was used to mask the retainer type using Blu-Mousse impression paste (Parkell, Edgewood, NY, USA) (Figure 4). Once applied, the impression paste was carefully trimmed with a scalpel blade to allow visualisation of the landmarks required for stability measurements. Placement of the impression paste was carried out before any model assessment by someone not involved in the trial.

## Statistical analyses

Descriptive statistics were used to describe the participants’ baseline characteristics, primary and secondary outcomes by randomisation group. The data were tested for normality. Mean (standard deviation) was reported if normally distributed, and median (interquartile range) was reported if not normally distributed.

The primary outcome was stability, measured by the change in Little’s Irregularity Index and intercanine width. This was compared using the Mann–Whitney *U* test.

Failure rates were analysed through survival analysis. Time to first failure of the retainer was compared between the groups using Kaplan–Meier plots and log-rank test. Hazard ratios (HR) and 95% confidence intervals (CI) were calculated for treatment groups using proportional hazard Cox regression. To assess the impact of risk factors on failure rate, the Cox model would have been further adjusted for recruiting site and clinical grade.

Patient satisfaction was reported using descriptive statistics.

All analyses were performed using R (version 3.4.0). The level of statistical significance was set as *P* < 0.05.

## Trial termination

Prior to trial commencement, it was agreed that the trial could be terminated if there was evidence that the interventions were causing harm to patients.

## Results

### Trial termination

The trial was terminated before reaching the full sample size due to the high number of failures in the upper Memotain^®^ retainer group (50% had some problem). As a result, the research team felt that they could not ethically continue the trial. From this point, no further patients were recruited to the trial. Patients who had been recruited to the trial but not yet had their six-month review were reviewed as normal. For patients already involved in the trial, at scheduled review appointments, vacuum-formed retainers were offered in addition to their bonded retainers.

The results of this study are therefore based on the findings from 62 patients. When considering the findings of this study, it is therefore important to consider that it is now underpowered and there is a risk of Type II error, meaning that the study fails to find a difference when one exists.

Consideration was given to continuing the trial and assessing only the lower bonded retainer. At the final research meeting it was clear, however, that both operators and participants had lost confidence in the Memotain^®^ retainer. Multiple operators commented on no longer feeling comfortable using Memotain^®^ retainers on their patients. Given the significant operator bias this would have introduced, the decision therefore was to stop the trial.

**Figure 5.**
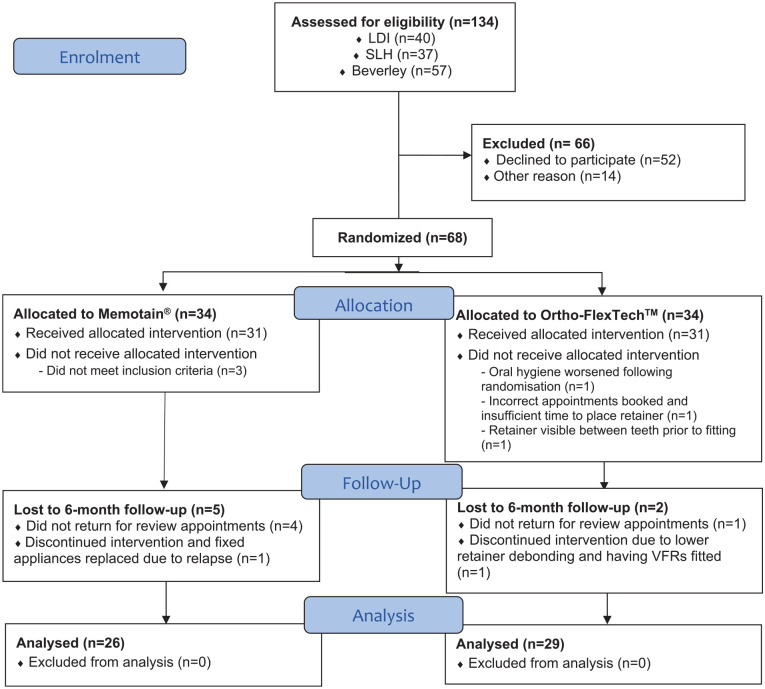
CONSORT flow diagram.

Figure 5 shows the CONSORT flow diagram for the study.

### Intra-rater reliability

Intra-class correlation (ICC) was used to assess the intra-rater reliability. The ICC values were all greater than 0.8 with the lower bound of 95% confidence intervals all greater than or equal to 0.75 indicating ‘good’ or ‘excellent’ reliability. The two lowest ICC reliability values were maxillary Little’s Irregularity Index (ICC = 0.893) and mandibular Little’s Irregularity Index (ICC = 0.909). The two highest ICC reliability values were maxillary intermolar width (ICC = 0.962) and mandibular intermolar width (ICC = 0.969).

### Baseline data

Baseline data are presented in Table 1 and show that there was equivalence between the two groups in terms site, grade of operator, irregularity at debond and intercanine width at debond. No difference in site or grade between groups illustrates effective randomisation and handling both known (and probably unknown) confounders well.

**Table 1.** Baseline characteristics at the time of fitting the retainer.

	Flex-Tech™	Memotain^®^	*P* value
n	29	26	
Recruiting site			0.759
Beverley	13 (44.8)	14 (53.8)	
Bradford	7 (24.1)	6 (23.1)	
Leeds	9 (31.0)	6 (23.1)	
Clinical grade			0.892
Consultant	7 (24.1)	7 (26.9)	
StR	9 (31.0)	9 (34.6)	
Therapist	13 (44.8)	10 (38.5)	
Upper retainer			
LII (mm)	0.39 (0.00–0.99)	0.00 (0.00–0.64)	0.139
ICW (mm)	33.90 ± 2.16	34.00 ± 1.84	0.846
Lower retainer			
LII (mm)	0.00 (0.00–0.39)	0.00 (0.00–0.26)	0.856
ICW (mm)	26.77 ± 1.95	26.44 ± 1.36	0.478

Values are given as n (%), mean ± SD or median (IQR).

ICW, intercanine width; LII, Littles Irregularity Index; IQR, interquartile range; SD, standard deviation.

### Survival

**Figure 6.**
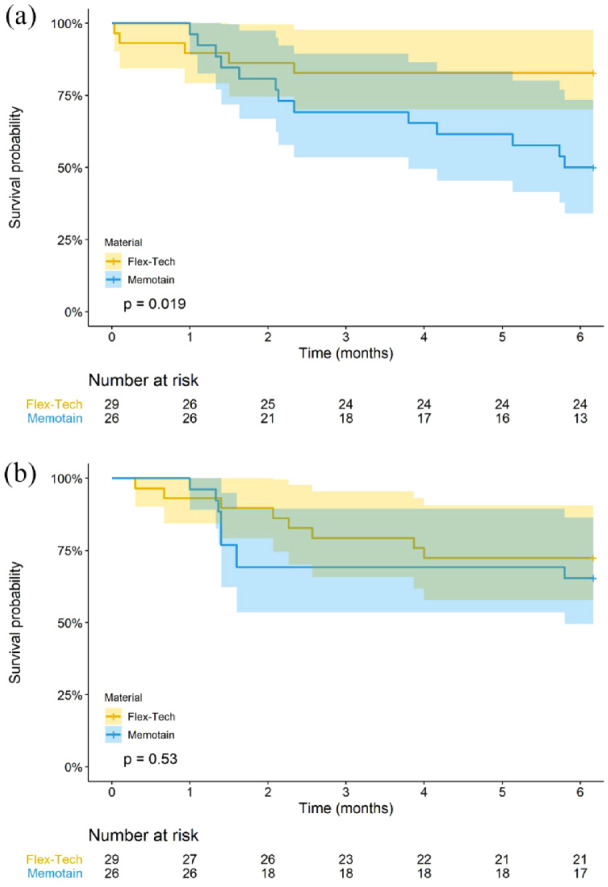
(a, b) Kaplan–Meier survival curve for the failure time of upper and lower retainers, for each retainer type.

Figure 6 shows the Kaplan–Meier plots for the time of first failure over the first six months.

Compared to Ortho-Flextech™, Memotain^®^ was approximately three times more likely to fail within six months (unadjusted HR = 2.82, 95% CI = 1.00-7.99, Table 2) in the upper arch. There was also a greater failure rate of Memotain^®^ than Ortho-Flextech™ for the lower retainer (unadjusted HR = 1.51, 95% CI = 0.57-3.99, Table 2), although not statistically significant.

**Table 2.** HR and 95% CI for upper and lower retainer failure at six months.

	HR (95% CI)			
	Upper retainer		Lower retainer	
	Unadjusted	Adjusted	Unadjusted	Adjusted
Material				
Flex-Tech™	1 (ref)	1 (ref)	1 (ref)	1 (ref)
Memotain^®^	3.22 (1.15–9.05)	2.82 (1.00–7.99)	1.37 (0.53–3.56)	1.51 (0.57–3.99)
Recruiting site				
Beverley		1 (ref)		1 (ref)
Bradford		0.99 (0.28–3.52)		2.95 (0.87–10.01)
Leeds		0.11 (0.01–1.39)		2.15 (0.38–12.36)
Clinical grade				
Consultant		1 (ref)		1 (ref)
StR		0.74 (0.12–4.46)		0.77 (0.16–3.79)
Therapist		0.43 (0.16–1.21)		1.07 (0.30–3.83)

CI, confidence interval; HR, hazard ratio.

Overall, the failure rates for Memotain^®^ and Ortho-Flextech™ were 50% and 17% for the upper retainer and 35% and 28% for the lower retainer, respectively, for those that were followed-up for six months (Figure 6).

With the available data, there was a lack of statistical power to show a significant effect on failure rates of the setting (University., Hospital or Specialist Practice) or grade of operator (Consultant Orthodontist, Orthodontic Registrar or Orthodontic Therapist). The number of failures (percentage) was reported for each factor by upper and lower retainers (Table 3).

**Table 3.** Number and percentage failures for each material, recruitment site and clinician grade.

	No. of failures (%)		
	Upper retainer	Lower retainer	Combined retainer
Material			
OrthoFlex-Tech™	5 (8.1)	8 (12.9)	13 (10.5)
Memotain^®^	13 (21.0)	9 (14.5)	22 (17.7)
Recruiting site			
Beverley	12 (19.4)	6 (9.7)	18 (14.5)
Bradford	5 (8.1)	6 (9.7)	11 (8.9)
Leeds	1 (1.6)	5 (8.1)	6 (4.8)
Clinical grade			
Consultant	8 (12.9)	4 (6.5)	12 (9.7)
StR	3 (4.8)	6 (9.7)	9 (7.3)
Therapist	7 (11.3)	7 (11.3)	14 (11.3)

Values are given as n (%).

**Figure 7.**
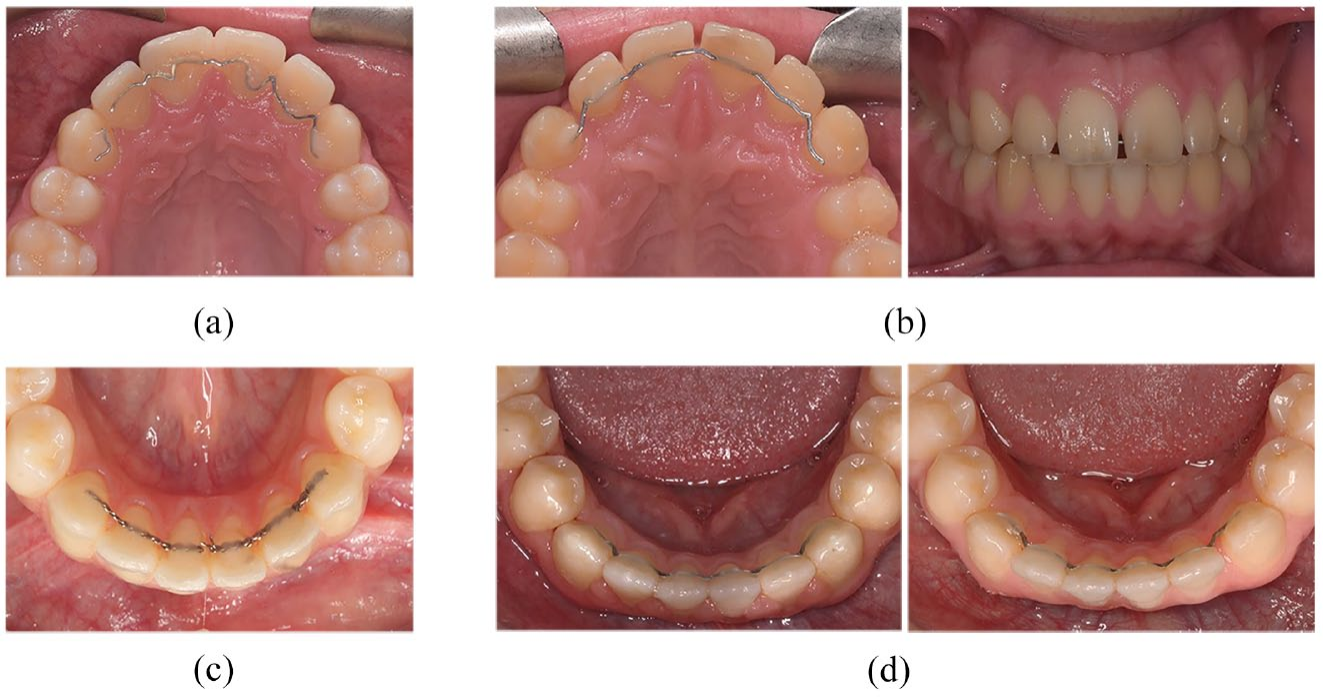
(a–d) Clinical photographs demonstrating some of the different reasons for bonded retainer failure for patients in the study. (a) Upper Memotain^®^ failure at six months – wire fracture between upper right lateral incisor and upper right canine. (b) Upper Memotain^®^ failure shown at debond and 6 months - composite/enamel interface failure upper left canine, composite/wire interface failure upper right canine with spaces opening and retainer visibility from the front. This participant required a second course of orthodontic treatment to address this. (c) Lower Ortho-FlexTech™ failure at 12 months – chain fracture between lower left central incisor and lower right central incisor. (d) Lower Memotain® failure shown at debond and 6 months - wire/composite interface failure with space opening between the lower left lateral incisor and canine. Composite/enamel interface failure seen on the lower right canine.

Figure 7 shows clinical photographs demonstrating examples of bonded retainer failures seen in the trial.

### Stability

**Figure 8.**
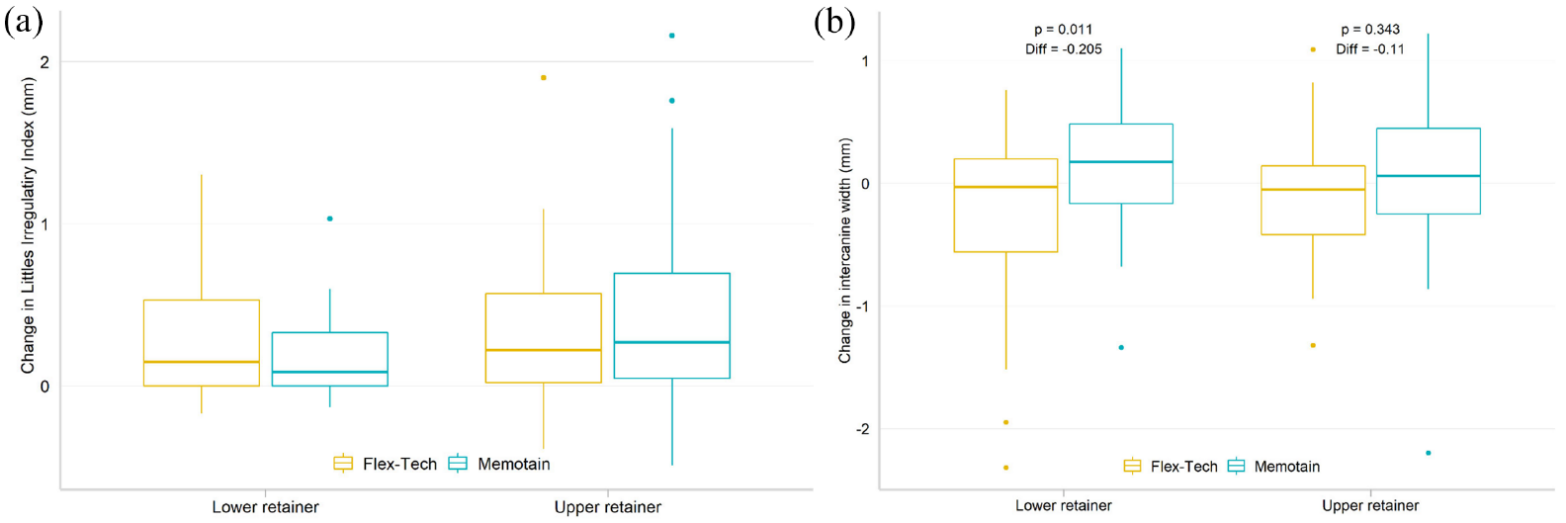
(a, b) Box plot for change in Little’s Irregularity Index and intercanine width between retainer fitting and six months follow-up, by fitting location and material. (a) Changes in Little’s Irregularity Index. (b) Changes in intercanine width.

The changes in irregularity, as measured with Little’s Index, are shown in Figure 8a.

Changes in intercanine width are shown in Figure 8b. In the lower arch, there was a statistically significant difference, with a slight increase in intercanine width of 0.2 mm with the Memotain^®^ retainer after six months.

**Figure 9.**
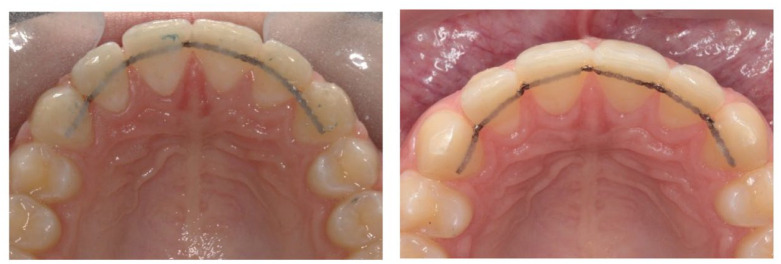
Upper Ortho-FlexTech™ relapse – retainer at debond and after six months, showing space opening distal to the lateral incisors, despite the retainer still being in place. This may reflect an increased interdental flexibility in this type of retainer, with a failure to cover the bonded retainer with adequate composite and excess bonded retainer chain between the teeth.

It was noted that even in cases where the retainer did not fail, relapse could still occur with the retainer in situ (Figure 9).

### Patient satisfaction

Table 4 shows the findings from the patient satisfaction questionnaires completed at the six-month review appointment. It was not appropriate to undertake statistical comparison of the retainers for patient satisfaction as the numbers of dissatisfied comments was low. It would appear that there was a high level of satisfaction with both types of retainers. Despite over 50% of upper Memotain^®^ retainer failures at six months, only 26% of the patients reported a problem that meant they felt they needed to see their orthodontist.

**Table 4.** Patient satisfaction with each retainer type at six months.

**Question on patient satisfaction questionnaire**	**Upper**				**Lower**			
	Memotain^®^ (n = 26)		Ortho-FlexTech™ (n = 29)		Memotain^®^ (n = 26)		Ortho-FlexTech™ (n = 29)	
	Yes	No	Yes	No	Yes	No	Yes	No
Did your retainer keep your teeth straight?	97%	3%	97%	3%	97%	3%	97%	3%
Was your retainer easy to look after?	100%	0%	100%	0%	100%	0%	97%	3%
Was your retainer comfortable?	94%	6%	100%	0%	100%	0%	100%	0%
Did your retainer affect your speech?	3%	97%	0%	100%	0%	100%	0%	100%
Did your retainer cause a problem that meant you needed to see your orthodontist?	26%	74%	10%	90%	13%	87%	13%	87%

## Discussion

### Termination of the study due to high failure rate of one retainer

The trial was terminated early due to the high number of failures noted with the Memotain^®^ retainers in the upper arch. The research team noticed early on that there appeared to be more failures in the upper arch. Concerns were raised by clinicians that they had seen relapse in some cases that would require retreatment. After discussion, it was decided that the failure rate would be reviewed when the first 50 patients recruited to the trial had been reviewed at six months. It was agreed that if a failure rate of 50% or more was noted, the trial would be halted. Although the protocol stated reasons for stopping the trial included harm to patients, this had not been defined with specific details. In future bonded retainer studies, it may be helpful to make a clear statement of stopping rules. A failure rate of 50% was noted with Memotain^®^ retainers in the upper arch; therefore, a decision was made by the team to stop further recruitment to the trial. In the protocol, no removable retainers were provided in addition to the bonded retainers, and there were concerns that this high level of retainer failure rate may result in high levels of relapse and the possible need for re-treatment in some patients. The failure rate for the Ortho-Flextech™ in the upper arch was 17%. It was noted that the risk of failure was about three times higher in the upper Memotain^®^ retainers than the Ortho-Flextech™ retainers. It is interesting to note that in both groups, approximately one-third of patients had failures in both arches, and two-thirds had failure only in one arch.

The most common site of failure with upper Memotain^®^ retainers was at the composite/enamel interface. It is not clear why this was the case. It is often presumed that failure at this interface could be related to poor clinical technique and moisture control; however, this is less of a problem in the upper arch. A standardised operating procedure, based on the manufacturer’s instructions, was used, and this was shared in training videos with the clinicians; however, it would appear that this was not sufficient to overcome a difference in clinical skills between operators. It is interesting that the use of precision-made CAD/CAM bonded retainer with a jig to help positioning of the wire and fitting of the wire did not seem to help. As noted in the methodology, there was a 10-day delay in the manufacturing and shipping of the retainer, so there is a theoretical risk of minor tooth movement with the fixed appliance in situ during this manufacturing period, which may have compromised the bond.

Upper bonded retainers are more likely to fail due to the effect of the occlusion. Despite the retainers being designed virtually with an occlusal registration, it is possible that some occlusal trauma increased the failure rate. Another possibility is that there is something inherent in the properties of the nitinol wire that makes it more likely to cause movement that compromises this bond.

Wire fractures were far less common in both groups for retainer failure, but it was more likely to occur in the Memotain^®^ group. This may be related to the more brittle nature of nitinol. The typical site of failure was between the upper canines and upper lateral incisors (Figure 7a).

There was no significant difference in the risk of failure in the lower retainers; however, the levels of failure were still relatively high at 35% (Memotain^®^) and 28% (Ortho-Flextech™) after six months. Failure at the composite/enamel interface was once again the most common site for retainer failure. These failure rates, although lower than the upper arch, are still disappointingly high. It is interesting that these higher failure rates are consistent with some other recent prospective controlled research (Naraghi et al, 2021; Wegrodzka et al, 2021), which tend to show higher failure rates than those measured in retrospective trials.

**Figure 10.**
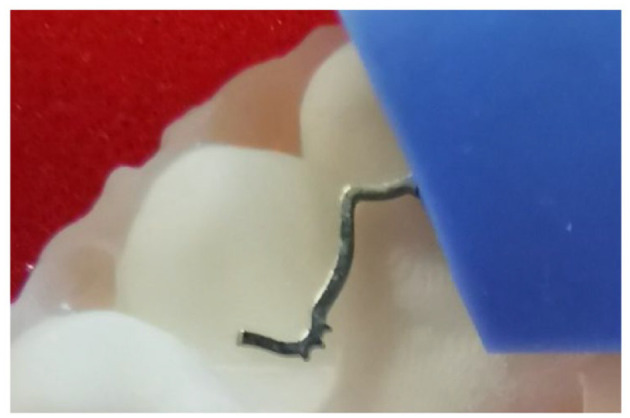
Memotain^®^ retainer showing finger-like projections over the canine teeth. This is a new feature of Memotain^®^ retainers designed to improve the bond between the wire and the composite. This new feature was not available in the version of retainers used in this trial.

One interesting example of failure in the lower Memotain^®^ group was between the wire and the composite interface (Figure 7b and 7d). This is a much less common site of failure of a bonded retainer. The consequence of failure at this interface resulted in space opening up. Composite does not chemically bond to metal, so this is usually a mechanical-type bond. As discussed earlier, the Memotain^®^ retainers are finished with an electropolishing process to produce a smooth finish to try and improve patient comfort. This smooth finish may be a disadvantage in the areas where the composite bonds with the metal. The manufacturers may have recognised this, and a newer version of the Memotain^®^ retainer (not used in this trial) has finger-like projections added in the areas where the composite will bond, which aims to improve the mechanical bond between the wire and the composite (Figure 10).

There was no statistically significant difference noted between the different settings or the different grades of operators (Table 2 and 3), which may reflect the sample size not being reached. Further research, with an appropriate sample size would be required to determine whether the different setting or grade of operator would affect the performance of bonded retainers.

It is worth noting that 52 participants declined to participate in the trial (Figure 5). The most common reason for this was patients not wanting to potentially delay their debond appointment and being randomised to the Memotain^®^ arm of the trial.

### Stability

It is interesting to note that despite the failure rates, the increase in irregularity, as measured with Little’s Irregularity Index, was minimal in the majority of cases. While there were isolated cases of clinically relevant relapse, in the majority of cases the retainers could be repaired or re-bonded before any unwanted tooth movement occurred. The main consequence of the retainer failures in this study was therefore the time, cost and inconvenience of repairing the retainer. This ranged from simply re-bonding an individual tooth back to the wire to complete replacement of the retainer on rare occasions. In the instances where spacing occurred, active appliances had to be placed for re-treatment (Figure 7b).

Stability was also assessed by measuring changes in intercanine width in both arches. Both types of retainers were relatively successful at maintaining the intercanine width. There was a slight increase in relapse in the intercanine width in the lower arch when using the Memotain^®^ retainer. However, this was exceptionally small—only a 0.2-mm difference after six months—which is not clinically significant.

### Patient satisfaction

It is difficult conclude with any degree of confidence which retainer type patients prefer, due to the very low levels of dissatisfaction with either retainer. It is perhaps safest to conclude that at six months, patients were satisfied with both types of retainers in terms of keeping the teeth straight, the retainers being easy to look after, being comfortable and not affecting speech.

Of patients, 26% reported that the upper Memotain^®^ retainers caused a problem that meant they needed to see their orthodontist. This is fewer than the number of patients who had failure of their upper retainer, suggesting that many of the failures went unnoticed by the patient, and highlighting the importance of regular bonded retainer review appointments within the first six months of placement. It would appear that it is not sufficient to presume that patients will recognise when there has been a failure of the bonding or integrity of the bonded retainer. If they are checked clinically, it may be possible to recognise and repair or replace a bonded retainer before any clinically significant relapse occurs.

### Cost-effectiveness not assessed in this trial

Due to the termination of the trial, we were unable to assess the cost-effectiveness of the two types of retainers. As one retainer is produced in advance via an external lab and the other is fitted directly at the chairside, there is a difference in the cost of the two retainers (with Memotain^®^ approximately 10 times more expensive). The amount of clinical time may also be different between the two retainers, which may also influence the overall cost. In future studies comparing lab-made retainers with retainers bonded directly at the chairside, it would be helpful to include cost-effectiveness as one of the outcomes.

### Comparisons to previous research

Previous randomised controlled clinical trials investigating Memotain^®^ have all been in the mandibular arch (Adanur-Atmaca et al., 2021; Alrawas et al., 2021; Kartal et al., 2021). The present study agrees with the findings of these studies, that the failure rates are lower in the mandibular arch, and levels of stability are good with mandibular retainers. This study provides new information on the use of Memotain^®^ in the upper arch, indicating that failure rates are much higher when used palatally in the upper labial segment.

There had previously been a shortage of high-quality research investigating Ortho-FlexTech™ bonded retainers, so this trial provides some useful information on stability, failure rates and patient satisfaction.

### Limitations

Due to the high number of problems with the upper Memotain^®^ retainers, the trial had to be stopped before the full sample size could be reached. It is therefore possible that the failure to show any difference between the retainers in some of the outcomes may be due to this smaller sample size. Interpretation of the findings should therefore be done with caution.

It was not possible to blind the operator or patient to the type of retainer placed, which may have introduced bias. However, the outcome assessor was successfully blinded.

Patient satisfaction is a complex area and can be difficult to measure. In this study, the questionnaire used the most relevant questions from a previous study (Forde et al., 2018). Further researchers may want to design and validate a questionnaire designed specifically for orthodontic retainers based on qualitative research.

The study was stopped after six months; therefore, it is important to note these findings are over a relatively short period. The information on problems of some of the retainers in the short term is certainly useful for clinicians. However, as retainers are now often recommended for long-term use, longer-term follow-up of retainers would be helpful.

### Generalisability

Previous studies investigating Memotain^®^ (Adanur-Atmaca et al., 2021; Alrawas et al., 2021; Kartal et al., 2021) have been undertaken in the University setting. This study was undertaken in a variety of settings (University, Hospital and Specialist Practice) and by different grades of clinicians (Consultant, Orthodontic Registrar and Orthodontic Therapist), which may make the findings more generalisable; however, the reduced sample size means the results should be viewed with caution. The wide range of confidence intervals in Table 2 does seem to suggest that individual variation is large, and the individual clinical skills of different operators may have a significant effect on the failure rates.

## Conclusion

After six months of maxillary and mandibular retention with Memotain^®^ and Ortho-FlexTech™ bonded retainers, the following conclusions can be drawn:

1. The trial had to be terminated early due to the high failure rate (50%) of upper Memotain^®^ bonded retainers within six months. The risk of failure was approximately three times higher than Ortho-FlexTech™ bonded retainers in the upper arch but the difference was not significantly different (HR, 95% CI).
2. There was no difference in the risk of retainer failure between Memotain^®^ bonded retainers and Ortho-FlexTech™ in the lower arch within six months.
3. There was a high overall patient satisfaction with both types of bonded retainers.

All of these conclusions are based on a reduced sample size due to the termination of the trial and should therefore be interpreted with caution.
